# Supplementary material for: Critical role for isoprenoids in apicoplast biogenesis by malaria parasites
Source: eLife. 2022 Mar 8;11:e73208. doi: 10.7554/eLife.73208 (PMC8959605; doi:10.7554/eLife.73208)
Supplement: Figure 3—source data 1. [file elife-73208-fig3-data1.pdf]

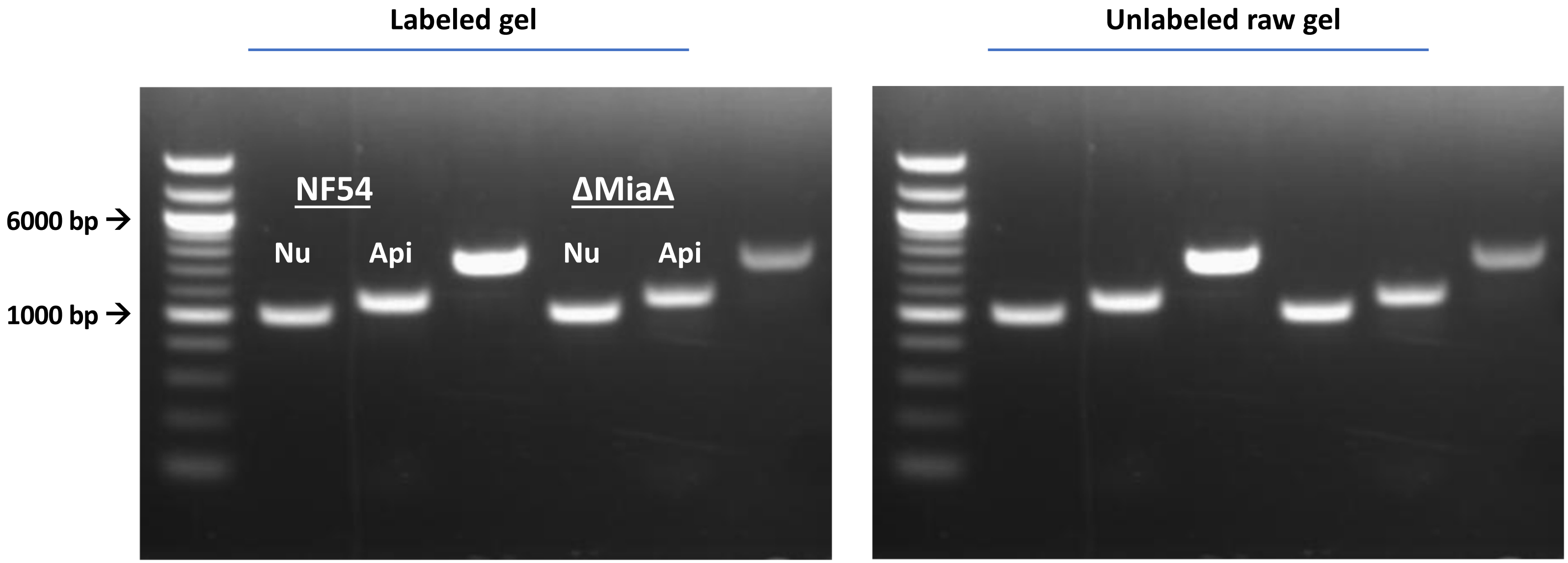

Figure 3- source data 1. Uncropped gel image of PCR analysis showing amplification of a (Nu) nuclear (PPS, PF3D7_0202700) and (Api) apicoplast (SufB, PF3D7_API04700) gene.
