# Supplementary material for: Critical role for isoprenoids in apicoplast biogenesis by malaria parasites
Source: eLife. 2022 Mar 8;11:e73208. doi: 10.7554/eLife.73208 (PMC8959605; doi:10.7554/eLife.73208)
Supplement: Figure 3—source data 2. [file elife-73208-fig3-data2.pdf]

**Labeled gel**

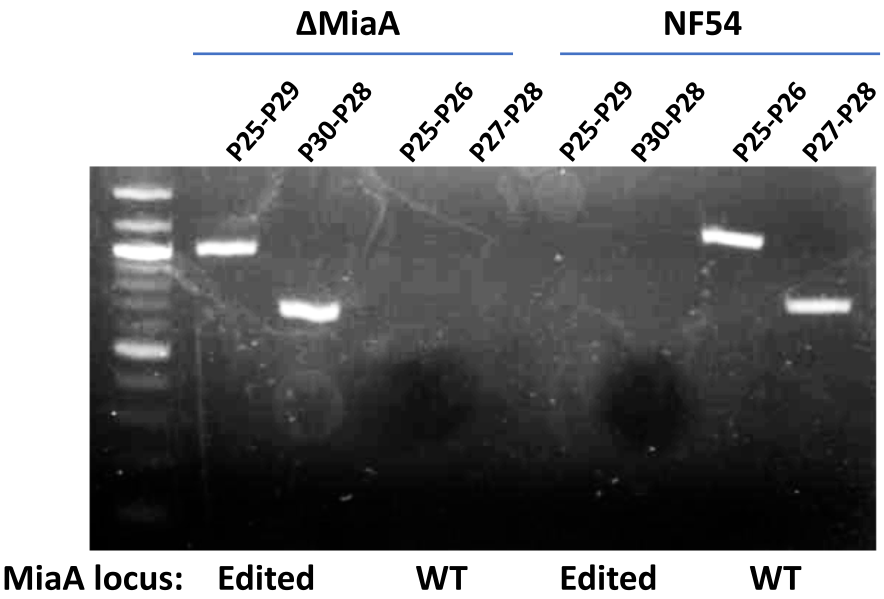


**Unlabeled raw gel**

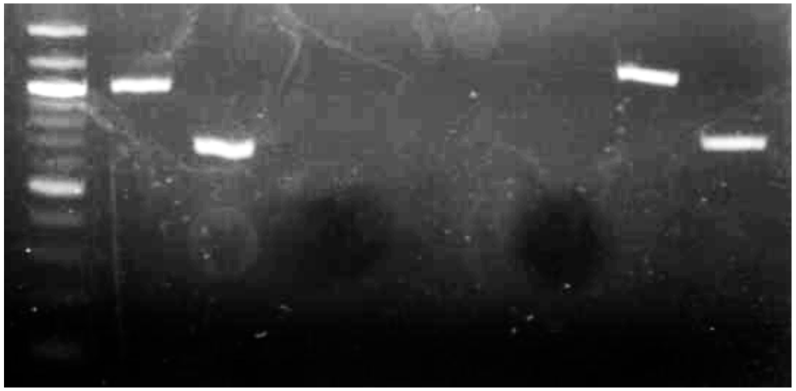

**Figure 3- source data 2.** Uncropped gel image of genomic PCR analysis of parental NF54 PfMev parasites and polyclonal transfected parasite progeny confirming successful disruption of MiaA.
