# Supplementary material for: Critical role for isoprenoids in apicoplast biogenesis by malaria parasites
Source: eLife. 2022 Mar 8;11:e73208. doi: 10.7554/eLife.73208 (PMC8959605; doi:10.7554/eLife.73208)
Supplement: Figure 5—source data 1. [file elife-73208-fig5-data1.pdf]

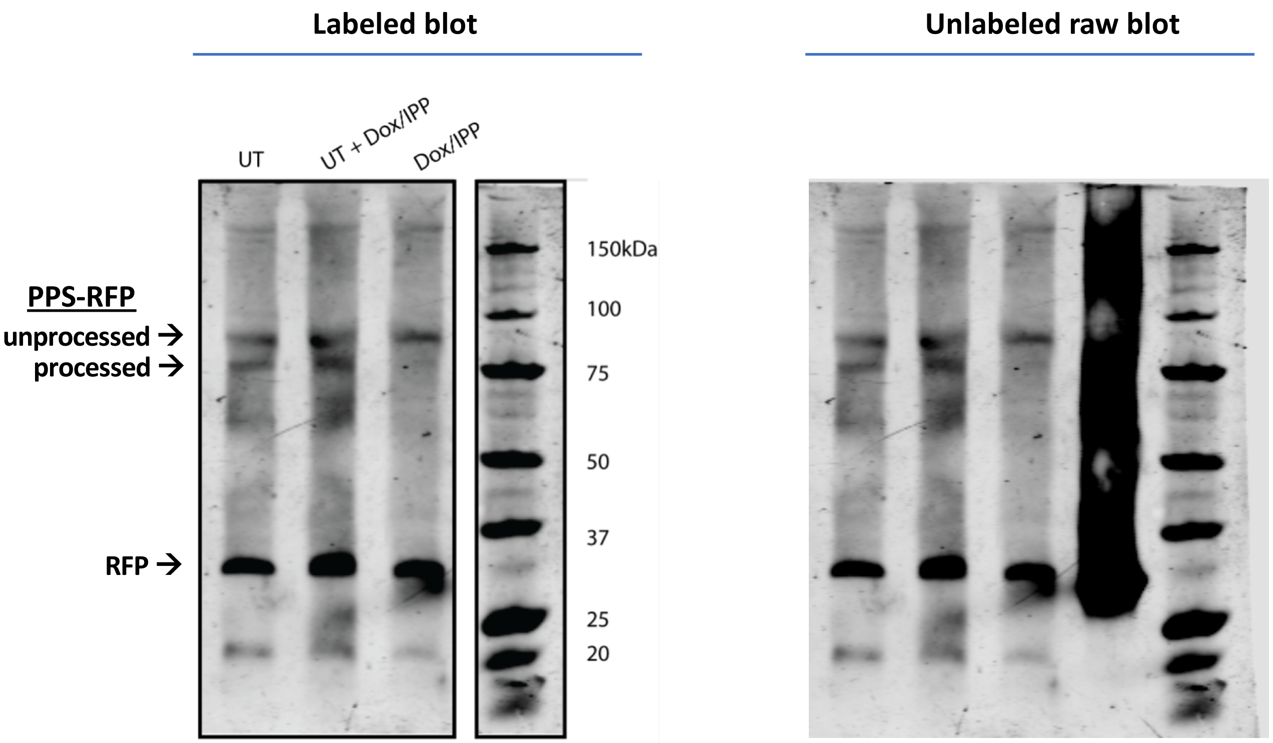

Figure 5- source data 1. Uncropped western blot image detecting PPS-RFP expression in Dd2 parasites. UT = untreated, Dox/IPP = parasites treated with 2 µM doxycycline and 200 µM IPP. The blot was probed with a mouse anti-RFP 1˚ antibody and donkey anti-mouse-DyLight800 2˚ antibody.
