# Supplementary material for: Critical role for isoprenoids in apicoplast biogenesis by malaria parasites
Source: eLife. 2022 Mar 8;11:e73208. doi: 10.7554/eLife.73208 (PMC8959605; doi:10.7554/eLife.73208)
Supplement: Figure 6—source data 1. [file elife-73208-fig6-data1.pdf]

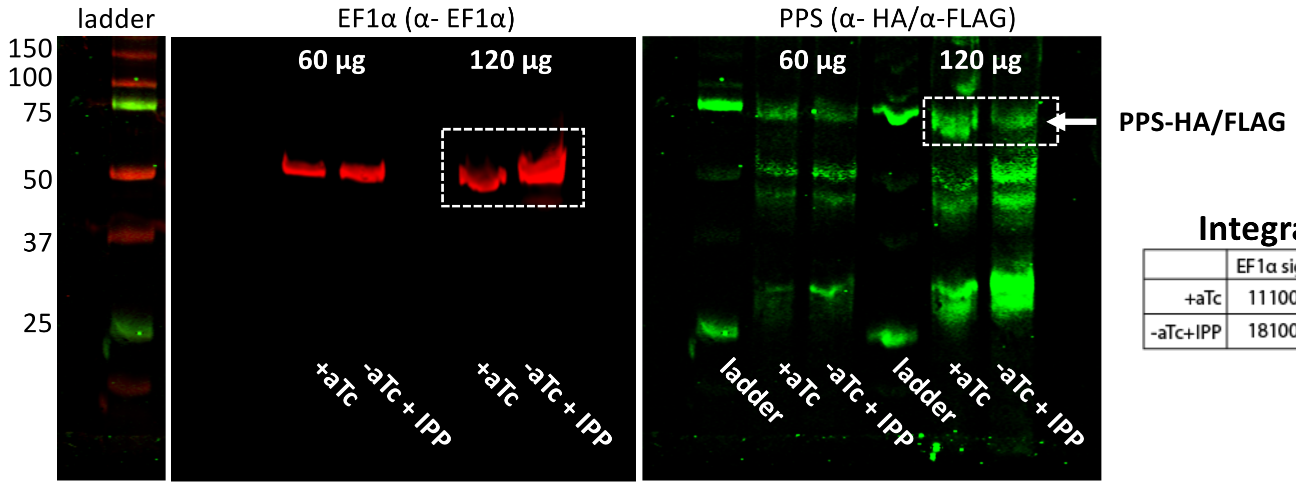

Integrated band intensities by densitometry

	EF1α signal	Normalization factor	PPS signal	Normalized PPS signal	+aTc / -aTc+IPP
+aTc	1110000	0.61	322	528	2.8
-aTc+IPP	1810000	1.00	187	187	

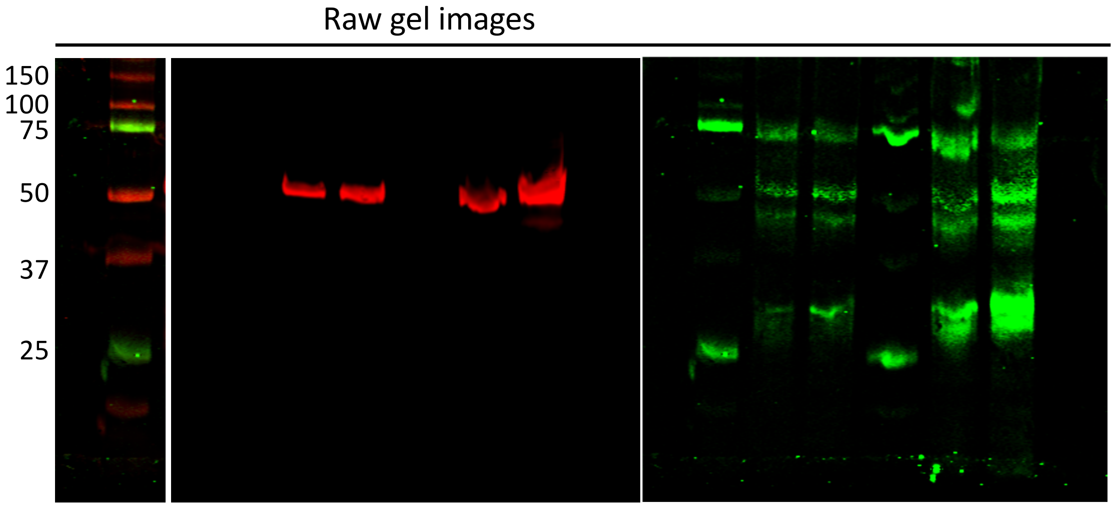

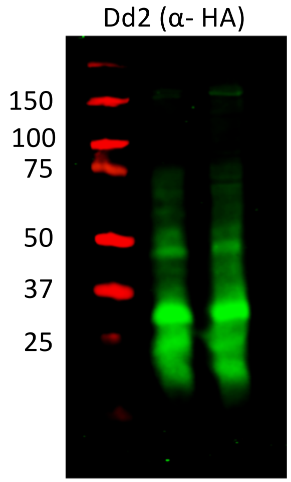

Figure 6- source data 1. Uncropped western blot images of Dd2 parasites with endogenously tagged PPS-HA/FLAG and probed with anti-HA and anti-FLAG antibodies and anti-EF1alpha antibody. The band for PPS was not detected in untagged parental Dd2 parasites.
