# Supplementary material for: Critical role for isoprenoids in apicoplast biogenesis by malaria parasites
Source: eLife. 2022 Mar 8;11:e73208. doi: 10.7554/eLife.73208 (PMC8959605; doi:10.7554/eLife.73208)
Supplement: Figure 6—source data 2. [file elife-73208-fig6-data2.pdf]

Labeled blot

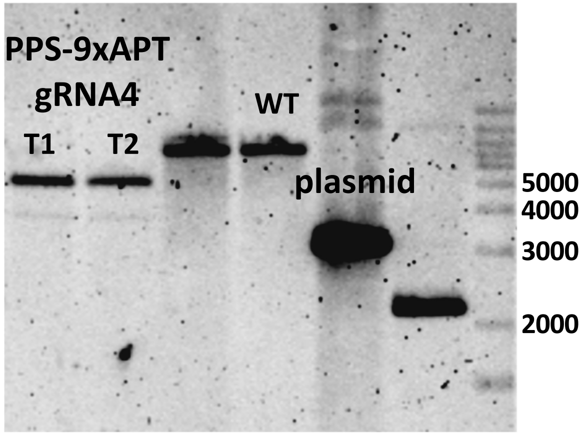

Unlabeled raw blot

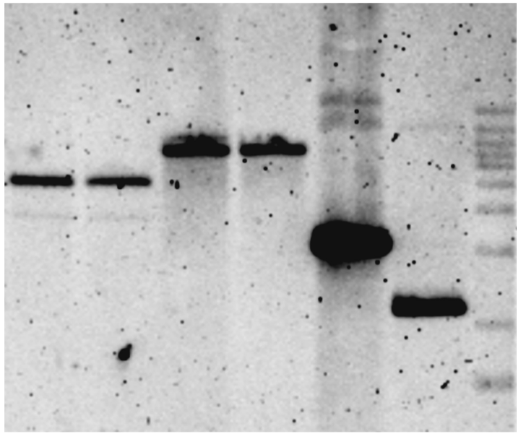

Figure 6- source data 2. Uncropped Southern blot image to probe integration of aptamer/TetR-DOZI cassette into PPS locus.
