# Supplementary material for: Critical role for isoprenoids in apicoplast biogenesis by malaria parasites
Source: eLife. 2022 Mar 8;11:e73208. doi: 10.7554/eLife.73208 (PMC8959605; doi:10.7554/eLife.73208)
Supplement: Supplementary file 1. [file elife-73208-supp1.docx]

| **Primers used in this study** | | |
| --- | --- | --- |
| **No.** | **Name** | **Sequence (5’ to 3’)** |
| 1 | PPS-EOE-F | ACACGATTTTTTCTCGAGATGGTTCACCTAAGTAAAAGAAATAATATTAAAAGCTTTTTA |
| 2 | PPS-GFP-TEOE-R | TGCTGCACCTGGCCTAGGTTTGACGTTTCTTGATAACACGTTTAAGATTAAATTAATTAA |
| 3 | PPS-RFP-TyEOE-R | TCAATTAAGTTTCCTAGGTTTGACGTTTCTTGATAACACGTTTAAGATTAAATTAATTAA |
| 4 | SufB-F | ACGATTTTTTCTCGAGATGATAAAATTAAAAAATTTTTTAAATATTTATAATTTAAATTA |
| 5 | SufB-R | TAGACACCATCCTAGGATTAAATATATCTTTAATTTTTAATGAAAATAATATAGGTATCT |
| 6 | PPS-Cas9gRNA1-F | TAAGTATATAATATTCGTGCTAGTTCTATTTTTGCGTTTTAGAGCTAGAA |
| 7 | PPS-Cas9gRNA1-R | TTCTAGCTCTAAAACGCAAAAATAGAACTAGCACGAATATTATATACTTA |
| 8 | PPS-Cas9gRNA2-F | TAAGTATATAATATTGATGATTCAAATAAAAGAAGGTTTTAGAGCTAGAA |
| 9 | PPS-Cas9gRNA2-R | TTCTAGCTCTAAAACCTTCTTTTATTTGAATCATCAATATTATATACTTA |
| 10 | PPS-Cas9gRNA4-F | TAAGTATATAATATT TGATATAAAACAAAGTAGCG GTTTTAGAGCTAGAA |
| 11 | PPS-Cas9gRNA4-R | TTCTAGCTCTAAAACCGCTACTTTGTTTTATATCAAATATTATATACTTA |
| 12 | Cas9HindIII-F | AATATTAAGCTTGTTTTAGAGCTAGAAATAGCAAGTTAAAATAAGGCTAGTCCGTTATCA |
| 13 | Cas9HindIII-R | TAAAACAAGCTTAATATTATATACTTAATATGAAATATGTGCATATAGGAAAAATTATGCATTTTGGTTACTCTAATATTATATATATAT |
| 14 | PPS_3’UTR_HF-F | GGCCCCTTTCCGGGCGCGCCCAATAACATATACAATATCAAACATATATATTATAATATTATTAAACATCTTCAATATTGTATTATTTAA |
| 15 | PPS_3’UTR_HF-R | TAATGCTATGACACCTCTTCTTTTATTTGACTTAAGTATGTTTGATACATGTAGATTTCTTAAAGAATGAAGCTTA |
| 16 | PPS_3’CR_HF-F | TTTAAGAAATCTACATGTATCAAACATACTTAAGTCAAATAAAAGAAGAGGTGTCATAGC |
| 17 | PPS_3’CR_HF-R | GTCATAAGGATAGACGTCATCATTTGACGTTTCTTGATAACACG |
| 18 | PPS_3’CR_HF-SM-R | CATAAGGATAGACGTCTTTGACGTTTCTTGATAACACGTTTAAGATTAAATTAATTAATGCTTCGCTACTTTGTTTTATATCATCATGTT |
| 19 | miaA.HA1.F | GCCACGAGCGGCCGTAAATTAAAGACAACGGGCTGTCAAC |
| 20 | miaA.HA1.R | AAGCGCAGCGGCCGGGAATTTCCATCTCTAAAAAAGTTCA |
| 21 | miaA.HA2.F | CGACAGACGCCGGTGAAAGAAATGATGATATGGTAGAATT |
| 22 | miaA.HA2.R | GGCCACCAGCCGGCGATATCCATCTTCTTTGTTTCTTGGC |
| 23 | miaA.gRNA.F | TAAGTATATAATATTAATAACGATATTAAATGTAAGTTTTAGAGCTAGAA |
| 24 | miaA.gRNA.R | TTCTAGCTCTAAAACTTACATTTAATATCGTTATTAATATTATATACTTA |
| 25 | miaA.5.F | GTTGAATAAATAAATGCCTCTCTATATATTGTTAACAT |
| 26 | miaA.5.WT.R | CTTCGACTTTAGCAATACCTACATTG |
| 27 | miaA.3.F | GGGAATCAGTAATTGATATAAGAAAAGAAG |
| 28 | miaA.3.WT.R | AAACTTCAAGACAATGCCTATAGC |
| 29 | pRS.F | CATATTTATTAAATCTAGAATTCGACAGACGCCGG |
| 30 | pRS.R | TACAAAATGCTTAAGCGCAGCGGCC |
| 31 | I5P-qPCR-F | GACATAAGTTTAGTAGGTCG |
| 32 | I5P-qPCR-R | TTCTGACTCCACATCATTTG |
| 33 | ADSL-qPCR-F | GGAAATCCATAGACAAACAATG |
| 34 | ADSL-qPCR-R | TCCTGTGAGAAGTGCTCCAC |
| 35 | TufA-qPCR-F | AAGATGTATTTTCTATAACAGGTAGAGGTA |
| 36 | TufA-qPCR-R | AACTGTTGTTAAATTAGGAGATGATTTTTC |
| 37 | PPS-qPCR-F | ATCAGGGGATTATCTCTTAGCAC |
| 38 | PPS-qPCR-R | AACTTTCGACAACATAAGAGAAACT |
| 39 | 3’APT-F | CTTATGACGTACCTGATTATGCAC |
| 40 | 10x APT-R | GTAGACCCCATTGTGAGTACATAAATATATTATATAAACTAGACTAGG |
