# Supplementary material for: Critical role for isoprenoids in apicoplast biogenesis by malaria parasites
Source: eLife. 2022 Mar 8;11:e73208. doi: 10.7554/eLife.73208 (PMC8959605; doi:10.7554/eLife.73208)
Supplement: Transparent reporting form [file elife-73208-transrepform1.docx]

***eLife’s* transparent reporting form**

We encourage authors to provide detailed information *within their submission* to facilitate the interpretation and replication of experiments. Authors can upload supporting documentation to indicate the use of appropriate reporting guidelines for health-related research (see [EQUATOR Network](http://www.equator-network.org/%20)), life science research (see the [BioSharing Information Resource](https://biosharing.org/)), or the [ARRIVE guidelines](http://www.plosbiology.org/article/info:doi/10.1371/journal.pbio.1000412) for reporting work involving animal research. Where applicable, authors should refer to any relevant reporting standards documents in this form.

If you have any questions, please consult our Journal Policies and/or contact us: [editorial@elifesciences.org](mailto:editorial@elifesciences.org).

**Sample-size estimation**

- You should state whether an appropriate sample size was computed when the study was being designed
- You should state the statistical method of sample size computation and any required assumptions
- If no explicit power analysis was used, you should describe how you decided what sample (replicate) size (number) to use

Please outline where this information can be found within the submission (e.g., sections or figure legends), or explain why this information doesn’t apply to your submission:

Sample replicate numbers were determined based on prior experience with the experiments that were performed and on practical considerations for carrying out these experiments. N numbers and statistical tests are described in the manuscript text, methods section, and figure legends.

**Replicates**

- You should report how often each experiment was performed
- You should include a definition of biological versus technical replication
- The data obtained should be provided and sufficient information should be provided to indicate the number of independent biological and/or technical replicates
- If you encountered any outliers, you should describe how these were handled
- Criteria for exclusion/inclusion of data should be clearly stated
- High-throughput sequence data should be uploaded before submission, with a private link for reviewers provided (these are available from both GEO and ArrayExpress)

Please outline where this information can be found within the submission (e.g., sections or figure legends), or explain why this information doesn’t apply to your submission:

We defined biological and technical replicates following the conventions of Blainey et al. (2014) *Nature Methods*, 11: 879-880. Biological replicates consisted of distinct parasite sample wells set-up and monitored in parallel for the duration of a given growth assay. Numbers of replicates for each experiment are given in the Methods section, in figure legends, and in Figure 1- Source Data 1 (for microscopy)

1. Growth assays (Figure 2B and 2C, Figure 8 supplement 1): Individual data points in plots are the average ± standard deviation of 3 biological replicates.

2. Growth assays (Figures 3A and 6E, Figure 4 supplement 1): Individual data points in plots are the average ± standard deviation of 3 biological replicates. No outlying data points have been excluded in plots.

3. Mass spectrometry experiments (Figure 8B): Beta-carotene levels are the average ± standard deviation of three biological replicates. No outlying data points have been excluded in plots.

3. Microscopy analysis (Figure 1C and 1E, Figure 1 supplement 2, Figure 4B, Figure 7C and 7E): Two independent experiments (biological replicates) were conducted, and 25 parasite images per replicate experiment were analyzed for each condition (50 total parasites counted for each condition). No outlying data was excluded. Raw parasite counts are given in Source data.

4. Microscopy analysis (Figure 2C and 2D, Figure 2 supplement 1-3): Cloning trays were performed from the growth assay shown in Figure 2B. 9 clones were expanded from 0hr+mev cloning tray, and 17-18 clones were expanded from the 30hr and 34hr+mev cloning tray. Only 5 clones returned in the 38hr +mev cloning tray. Two images were taken for each clone, and all imaged clones are shown in Figure 2 supplement 1-3. No outlying data was excluded.

5. Microscopy analysis (Figure 3B). 10 parasites from each condition were observed with 4 images reported. No outlying data was excluded.

6. qPCR analysis (Figure 6C and 7F): qPCR ratios were normalized to +aTc in each case and are the average ± standard deviation of three biological replicates. No outlying data was excluded.

**Statistical reporting**

- Statistical analysis methods should be described and justified
- Raw data should be presented in figures whenever informative to do so (typically when N per group is less than 10)
- For each experiment, you should identify the statistical tests used, exact values of N, definitions of center, methods of multiple test correction, and dispersion and precision measures (e.g., mean, median, SD, SEM, confidence intervals; and, for the major substantive results, a measure of effect size (e.g., Pearson's r, Cohen's d)
- Report exact p-values wherever possible alongside the summary statistics and 95% confidence intervals. These should be reported for all key questions and not only when the p-value is less than 0.05.

Please outline where this information can be found within the submission (e.g., sections or figure legends), or explain why this information doesn’t apply to your submission:

Descriptions of statistical tests are given in Methods and in figure legends. For experiments involving repeat measurements, an average ±standard deviation is reported. Differences in averages from repeat measurements were analyzed for significance by two-tailed unpaired t-test. P values for all such tests are explicitly stated in all figures.

(For large datasets, or papers with a very large number of statistical tests, you may upload a single table file with tests, Ns, etc., with reference to sections in the manuscript.)

**Group allocation**

- Indicate how samples were allocated into experimental groups (in the case of clinical studies, please specify allocation to treatment method); if randomization was used, please also state if restricted randomization was applied
- Indicate if masking was used during group allocation, data collection and/or data analysis

Please outline where this information can be found within the submission (e.g., sections or figure legends), or explain why this information doesn’t apply to your submission:

For the microscopy analysis in Figure 1C/E, Figure 1 supplement 2, Figure 2C/D and figure supplement 1-3, Figure 4B, and Figure 7C/E, apicoplast status was scored as normal, punctate or disrupted. Normal apicoplast morphology was determined by the presence of apicoplast elongation or branching. Punctate was determined by the presence of a single focal apicoplast signal (in trophozoites or schizonts). Disrupted apicoplast status was determined by the presence of numerous, scattered apicoplast signals, which were not coordinated to any observed nuclei.

**Additional data files (“source data”)**

- We encourage you to upload relevant additional data files, such as numerical data that are represented as a graph in a figure, or as a summary table
- Where provided, these should be in the most useful format, and they can be uploaded as “Source data” files linked to a main figure or table
- Include model definition files including the full list of parameters used
- Include code used for data analysis (e.g., R, MatLab)
- Avoid stating that data files are “available upon request”

Please indicate the figures or tables for which source data files have been provided:

All data is shown in figures and supplemental figures in our manuscript. Uncropped gel or western blot images are provided as source data for Figure 2C, Figure 3B, Figure 3- figure supplement 1, Figure 5E, Figure 6C, and Figure 6- figure supplement 1. Parasite counts for all microscopy analyses are provided as source data.
